# Lessons for Developing Countries From Outlier Country Health Systems

**DOI:** 10.3389/fpubh.2022.870210

**Published:** 2022-06-22

**Authors:** Nachiket Mor

**Affiliations:** The Banyan Academy of Leadership in Mental Health, Thiruvidanthai, India

**Keywords:** out-of-pocket expenditures (OOPE), Disability Adjusted Life Year (DALY), financial protection, universal health care (UHC), total health expenditure

## Abstract

Building good health systems is an important objective for policy makers in any country. Developing countries which are just starting out on their journeys need to do this by using their limited resources in the best way possible. The total health expenditure of a country exerts a significant influence on its health outcomes but, given the well-understood failures of price-based market-mechanisms, countries that spend the most money do not necessarily end-up building the best health systems. To help developing country policy makers gain a deeper insight into what factors matter, in this study the contribution of per-capita total, out-of-pocket, and pooled health expenditures, to the cross-country variation in Disability Adjusted Life Years lost per 100,000 population (*DALY Rates*), a summary measure of health outcomes, is estimated. The country-specific residuals from these analyses are then examined to understand the sources of the rest of the variation. The study finds that these measures are able to explain between 40 and 50% of the variation in the *DALY Rates* with percentage increases in per-capita out-of-pocket and pooled expenditures being associated with improvements in *DALY Rates* of about 0.06% and 0.095%, respectively. This suggests that while increases in per-capita total health expenditures do matter, moving them away from out-of-pocket to pooled has the potential to produce material improvements in *DALY Rates*, and that taken together these financial parameters are able to explain only about half the cross-country variation in *DALY Rates*. The analysis of the residuals from these regressions finds that while there may be a minimum level of per-capita total health expenditures (> $100) which needs to be crossed for a health system to perform (Bangladesh being a clear and sole exception), it is possible for countries to perform very well even at very low levels of these expenditures. Colombia, Thailand Honduras, Peru, Nicaragua, Jordan, Sri Lanka, and the Krygyz Republic, are examples of countries which have demonstrated this. It is also apparent from the analysis that while very high rates (> 75%) of pooling are essential to build truly high performing health systems (with *DALYRates* < 20, 000), a high level of pooling on its own is insufficient to deliver strong health outcomes, and also that even at lower levels of pooling it is possible for countries to out-perform their peers. This is apparent from the examples of Ecuador, Mexico, Honduras, Malaysia, Vietnam, Kyrgyz Republic, and Sri Lanka, which are all doing very well despite having *OOP%* in the region of 40–60%. The analysis of residuals also suggests that while pooling (in any form) is definitely beneficial, countries with single payer systems are perhaps more effective than those with multiple payers perhaps because, despite their best efforts, they have insufficient market power over customers and providers to adequately manage the pulls and pressures of market forces. It can also be seen that countries and regions such as Honduras, Peru, Nicaragua, Jordan, Sri Lanka, Bangladesh, Kerala, and the Kyrgyz Republic, despite their modest levels of per-capita total health expenditures have delivered attractive *DALY Rates* on account of their consistent prioritization of public-health interventions such as near 100% vaccine coverage levels and strong control of infectious diseases. Additionally, countries such as Turkey, Colombia, Costa Rica, Thailand, Peru, Nicaragua, and Jordan, have all delivered low *DALY Rates* despite modest levels of per-capita total health expenditures on account of their emphasis on primary care. While, as can be seen from the discussion, several valuable conclusions can be drawn from this kind of analysis, the evolution of health systems is a complex journey, driven by multiple local factors, and a multi-country cross-sectional study of the type attempted here runs the risk of glossing over them. The study attempts to address these limitations by being parsimonious and simple in its approach toward specifying its quantitative models, and validating its conclusions by looking deeper into country contexts.

## 1. Introduction

**Figure 1:**
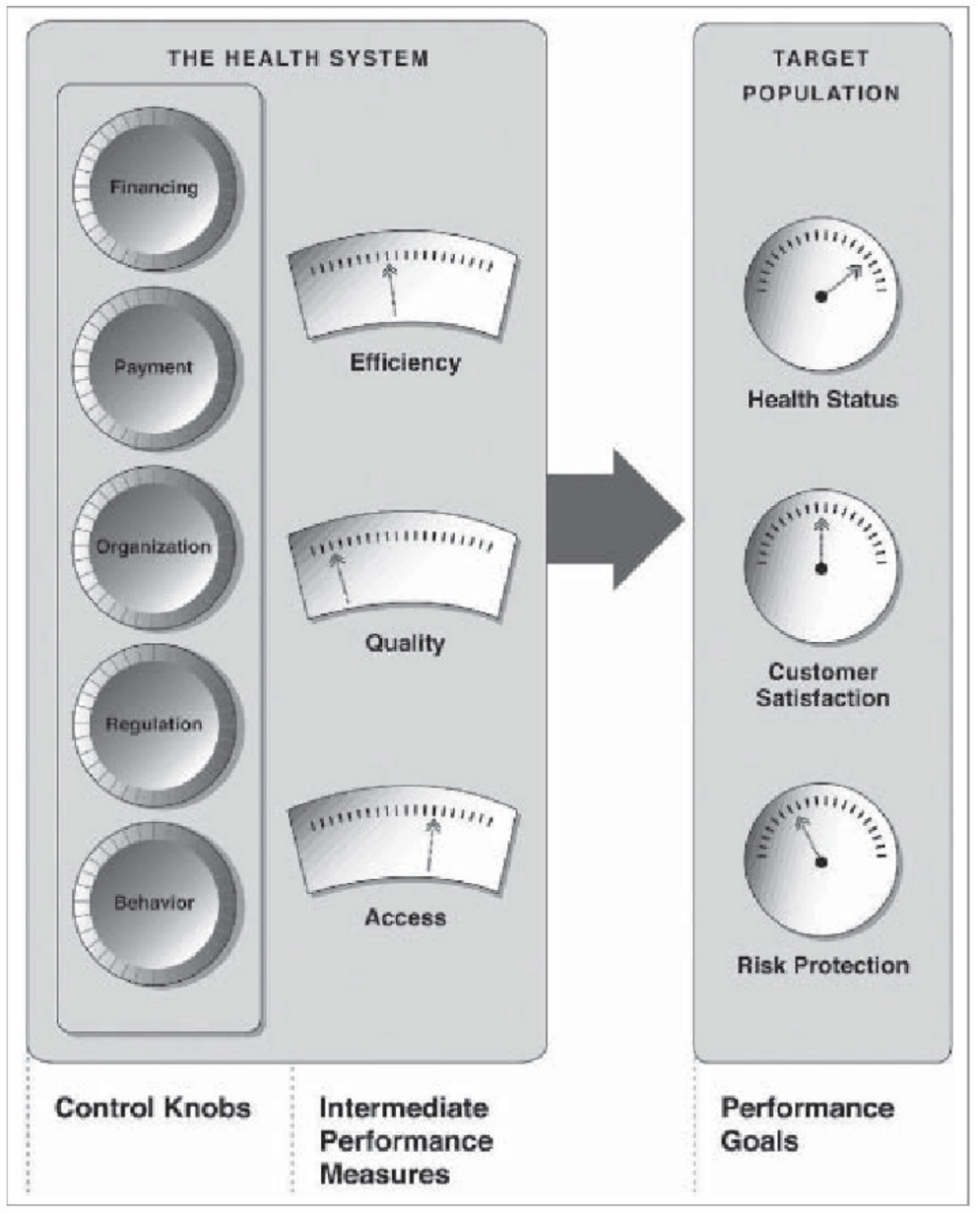
Control Knobs Framework (1).

Building good health systems in an efficient way is an important objective for policy makers in any country, but in particular for those in developing countries which are just starting out on their journeys, and need to use their limited resources in the best way possible. An important framework that is used to guide policy formulation is the Control Knobs Framework shown in Figure 1. As can be seen from the figure, the framework takes the view that there are multiple Control Knobs that can be dialled up or down by policy makers to reach their desired health systems goals. One of the key Control Knobs is that of financing which refers to, among other things, the total quantum of funds that are spent on healthcare in a county, and manner in which they are spent (1). These questions are often starting points for any analysis of health systems.

The per-capita health expenditure of a country is determined by its per-capita income and by the proportion of that income that is allocated toward healthcare by its citizens and governments, and has a significant influence on health outcomes. However, given the well-understood failures of price-based market-mechanisms to build good health systems (2), countries that spend the most money do not necessarily end-up building the best health systems. Within the financing domain, one such set of added factors which are considered to be important (3, 4) relate to the extent to which the health expenditures in a country are pooled, and how much citizens are required to spend on an out-of-pocket basis, when they seek healthcare. That all of these factors matter is a widely accepted view, however, from a policy perspective it would be important get a more precise estimate of the extent of their importance. Additionally, once a careful determination has been made of the extent to which these two aspects of financing matter, it then becomes important to understand what role, if any, the other Control Knobs have in influencing the performance of health systems. The study therefore attempts to explore three broad questions:

1. To what extent are countries that have higher per-capita health expenditures able to generate better health outcomes?
2. To what extent is pooling of these expenditures important?
3. Are there factors, other than per-capita health expenditures and the extent of pooling, which have an impact on health outcomes?

## 2. Data

To carry out these analyses, data are needed for health systems performance, total heath expenditures, out-of-pocket expenditures, and pooled expenditures, for countries and regions around the world. All of the data used in this study have been reported in **Tables 11**–**15**.

In this study Disability Adjusted Life Years lost (DALYs), as defined by the Institute for Health Metrics and Evaluation, (5), per 100,000 population, referred to as the *DALY Rate*, is used as the measure of health status (Figure 1).^1^ For countries data on *DALY Rates* have been obtained from the Institute for Health Metrics and Evaluation (7). In addition, given their sheer sizes, the wide variations between them in both inputs and outputs, and the constitutional authority and resources possessed by them to manage their own health systems, Indian states, for which data are available, are treated on par with independent countries, and India as a country is omitted from the data set. The *DALY Rates* for Indian states have been obtained from the statistical appendix to the Global Burden of Disease Study for Indian states (8, 9).

It is important to note that the *DALY Rates* used in this study are **not** age-standardized. This is because population-age distributions vary widely between countries, as exemplified by the variation in the proportion of 0–14 year olds across countries (10). These variations, in a manner similar to other environmental variables such as distance from the equator (11), have a material impact how much countries spend on their health systems, how they design them, as well as on the performance of these health systems. The question being asked in this paper is, given the reality in which countries find themselves, how well have they responded through their health systems. Age standardizing only the *DALY Rates* and not the inputs into the health system, would end-up systematically biasing the results in favor of (against) health systems of the more developed countries (less developed countries) which have a higher (lower) proportion of older individuals.

It is also important to bear in mind that the use of *DALY Rate* to characterize the overall performance of health systems, does not directly assess the performance of these systems on other factors listed in Figure 1, such as the degree of financial protection offered, equity of access, and degree of responsiveness. While, if poor performance of the health system on any of these factors is sufficiently large it is possible that it would be reflected in the *DALY Rate*, a proper study of what drives the performance of health systems on these factors would need an entirely different approach—a cross-sectional analysis of the type undertaken here may not be best suited to study the drivers of performance of health systems on these dimensions.

Data on per-capita total health expenditures (*THE*) for countries have been obtained from the data sets published by the World Bank (12). For the Indian states *THE* data have been obtained from the 2015-16 National Health Accounts estimates published by the National Health Systems Resource Centre (13). Sufficient data could not be obtained for a number of smaller countries, including American Samoa, Aruba, Bermuda, British Virgin Islands, Greenland, Guam, North Korea, Kosovo, Libya, and Hong Kong, and within India, for the state of West Bengal. All of these countries and regions have therefore been omitted from the analysis. The *THE* data have all been expressed in US Dollars, measured using Purchasing Power Parity (*PPP*) exchange rates (14). An exchange rate of Rs. 18.55 per US$ has been used in the study to convert state level rupee expenditures into PPP US Dollar numbers for the Indian states.

**Figure 2:**
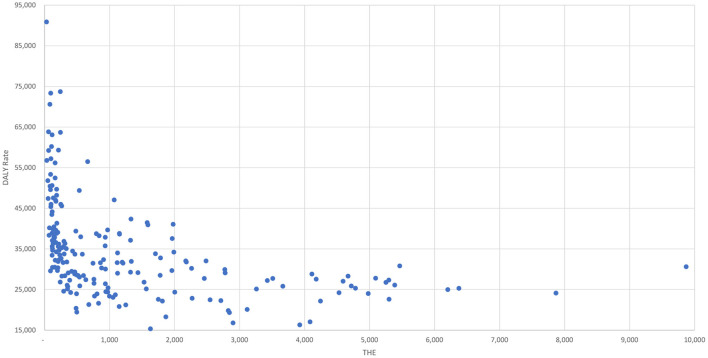
DALY Rate vs. THE for 2016.

Using 2016 data, Figure 2 graphs the relationship between the *DALY Rates* and *THE* for all the countries (and Indian states) in the data-set. It can be seen from the graph that, while at lower levels of *THE* even small increases are associated with large reductions in the *DALY Rate*, the benefits that accrue from additional increases in *THE* appear to decline at an exponential rate.

The 2016 data on per-capita out-of-pocket expenditures (*OOP*) for countries have been obtained from the data sets published by the World Bank (15). For the Indian states the 2016 *OOP* data have been obtained from the 2015-16 National Health Accounts estimates published by the National Health Systems Resource Centre (13). Per-capita Pooled Expenditures (*POOL*) for each country (*i*), are computed simply as:


(1) $$\begin{array}{r} POOL_{i}=THE_{i}-OOP_{i} \end{array}$$

While population data are not used directly in any of the quantitative analyses in the study, they are included in the tables to provide an assessment of the overall size of the country. For countries population numbers have been obtained from data published by the World Bank (16). For Indian States the population numbers, given the recent separation of the state of Telangana from the state of Andhra Pradesh, the only source from which 2016 population data could be obtained was the website IndiaPopulation2019 (17).

## 3. Methods

The study attempts to quantify the impact of *THE, OOP*, and *POOL* on the *DALY Rate* using multi-country cross-sectional analysis. Multi-country cross-sectional analysis of this sort is fraught with a number of difficulties and has been famously compared by Joan Robinson to looking for a black cat in a dark room where no cat exists (18). There are also a number of continuing questions about the methodology behind estimating *DALYs* which limit the value of attempting to obtain more precise and detailed conclusions from further cross-country quantitative data-analysis (19–21). Despite these concerns, given the salience of these variables, there is value in examining the degree to which they have explanatory power, and what lessons they hold for policy makers. However, given these issues, once the analysis of the relationship between *THE, OOP, POOL*, and *DALY Rates* is complete, instead of attempting further quantitative analyses to explain the rest of the variation in *DALY Rates*, a comparison of the predicted values of health outcomes with actuals, is used in the study to identify positive and negative outlier countries. The unusual performance of these outliers is then explored further, to see if there are additional lessons to be learnt from them for wider application.

The exponential relationship between the *DALY Rate* and *THE* is apparent from Figure 2 and suggests that a simple Cobb-Douglas production function (22) as shown in Equation (2), could be used to explore the relationships between these variables for each country *i*, and to understand how much of the variation in the *DALY Rates* they are able to explain—the variable labeled *DALY* in the equations refers to the *DALY Rate*.


(2) $$\begin{array}{r} DALY_{i}=Q_{i}THE_{i}^{\gamma } \end{array}$$

(3) $$\begin{array}{r} \;\Rightarrow \;\ln DALY_{i}=\ln Q_{i}+\gamma \ln THE_{i} \end{array}$$

with γ being the elasticity of percentage change in the *DALY Rate* for every percentage change in *THE* and ln *Q*_*i*_ being the portion not explainable by the changes in *THE*.

For estimation purposes this equation may be rewritten as:


(4) $$\begin{array}{r} \ln DALY_{i}=\ln Q_{0}+\gamma \ln THE_{i}+\ln\eta_{i} \end{array}$$

where, η_*i*_ is the residual or the error term for each country *i*, which captures the unexplained part of the performance of each health system, with ln *Q*_0_ being the constant term (i.e., ln *Q*_*i*_ = ln *Q*_0_ +ln η_*i*_).

*THE* can be further divided into Pooled Expenditures (*POOL*) and Out-of-Pocket Expenditures (*OOP*) incurred by the consumers of healthcare services. Since *POOL* and *OOP* evolve independently in any health system, using these variables, Equation (2) may be rewritten as:


(5) $$\begin{array}{r} DALY_{i}=Q_{i}POOL_{i}^{\alpha }OOP_{i}^{\beta } \end{array}$$

where, α and β are the elasticities associated, respectively, with *POOL* and *OOP*.

Just as has been done in Equation (4), the associated regression equation may be written as:


(6) $$\begin{array}{r} \ln DALY_{i}=\ln Q_{0}+\alpha \ln POOL_{i}+\beta \ln OOP_{i}+\ln\epsilon_{i} \end{array}$$

Having estimated these elasticities, the residual associated with each country *i*, $\hat{\epsilon_{i}}$ is estimated as:


(7) $$\begin{array}{l} \hat{\epsilon_{i}}=DALY_{i}-\hat{DALY_{i}}\\ \;=DALY_{i}-\exp(\hat{\ln Q_{0}}+\hat{\alpha }\ln POOL_{i}+\hat{\beta }\ln OOP_{i}) \end{array}$$

where, $\hat{\ln Q_{0}}$, $\hat{\alpha }$, and $\hat{\beta }$ are the estimated values from Equation (6).

These residuals (ϵ_*i*_), reported in **Tables 11**–**15**, which represent the extent to which the health system is an **outlier** (i.e, has aspects of its performance that are not explainable by *POOL* and *OOP*) are then subjected to further examination to ascertain if they offer any lessons for developing country policy makers.

## 4. Results and Findings

### 4.1. Role of Total Health Expenditures (*THE*)

Using this data, Equation (4) is estimated using Ordinary Least Squares (OLS). The results of this regression are given in Table 1. These results suggest that with an elasticity γ = −0.1557 (*p* << 0.01%) applied to *THE*, it is possible to explain 49.43% of the variation in *DALY Rates*, leaving fairly large unexplained residuals. The large value of ln*Q*_0_ can perhaps be seen as the centrifugal force (23) pulling country health systems toward a baseline level of low-performance.^2^ The large value of ln*Q*_0_ implies that $Q_{0}=e^{\ln Q_{0}}=89,035.36$, a DALY Rate comparable to that of the Central African Republic (see **Table 8**). The value of γ = −0.1557 also indicates that countries and regions desirous of bringing down *DALY Rates* by 15.57% would need to double their *THE* and that a halving of the *DALY Rate* would need a tripling of total health expenditures. It is clear from the analysis that *THE* is an important driver of *DALYs* but increases of the magnitude required would necessarily have to follow the natural growth curve of the per-capita incomes in these economies which, in turn, would need them to allocate increased amounts in the development of their own Human Capital (25), money that they may not necessarily be able to find, at least in the near term. These limitations make it important to examine if, even within the current levels of *THE* there are other opportunities that developing country policy makers have, to generate a positive impact on *DALY Rates*.

**Table 1:** Regression results with *THE*.

**Variable**	**Coefficients**	**Standard error**	**t stat**	***P*-value**
ln*Q*_0_	11.3968	0.1005	113.43	3.19E-103
γ	−0.1557	0.0162	−9.64	9.91E-016
*R* ^2^	0.4943	0.2134		

### 4.2. Role of Pooled Expenditures (*POOL*)

As a next step, using 2016 data, when Equation (6) is estimated using Ordinary Least Squares (OLS) the results given in Table 2 are obtained.

**Table 2:** Regression results with *POOL* and *OOP*.

**Variable**	**Coefficients**	**Standard error**	**t stat**	***P*-value**
ln*Q*_0_	11.2445	0.0728	154.47	2.32E-203
α	−0.0941	0.0153	−6.15	4.50E-009
β	−0.0584	0.0173	−3.38	8.92E-004
*R* ^2^	0.4322	0.2365		

These results suggest that with an elasticity of −0.0941 applied to *POOL* and −0.0584 applied to *OOP*, it is possible to explain 43.22% of the variation in the *DALY Rates* leaving, once again, fairly large unexplained residuals. It can be seen from the estimated values of α and β that, as expected, pooled expenditures have a far greater impact on reducing *DALYs* than do out-of-pocket expenditures. This differential in elasticities suggests a potentially additional pathway toward improving the performance of health systems, and one that is much more directly in the hands of the government than is increasing *THE*, i.e., increasing the quantum of pooled expenditures and reducing the amounts being spent on an out-of-pocket basis. Given the relative values of α and β, for a region (like the Indian state of Kerala, for example) where the *OOP%* ≈ 70%, a 10% reduction in the level of *OOP* would improve the *DALY Rate* by 0.584%. However, if that reduction in *OOP* is entirely reallocated to pooled expenditures, it would result in a 21% increase in *POOL* and consequently a 1.98% reduction in DALYs. This represents a net improvement of 1.4% in the *DALY Rate* without any increase in *THE* itself. These reallocations are hard to accomplish but are likely to be easier than pushing per-capita growth rates in the entire economy and *THE* to higher levels.

Driven by this insight [using *OOP%* data from (26)], and other considerations relating to financial protection and equity (Figure 1), it can be seen from Table 3 that many countries, have reduced the *OOP%* between the years 2016 and 2000, in order to improve the performance of their health systems.

**Table 3:** Countries with > 20% reduction in OOP% from 2000 to 2016.

**Country**	**ΔOOP%**	**OOP%**	**OOP%**
		**2000**	**2016**
Maldives	43.89	62.99	19.10
Mali	38.45	73.73	35.28
Sierra Leone	34.10	75.65	41.55
Gabon	30.62	53.13	22.51
Mauritania	30.13	81.03	50.90
Lebanon	25.63	57.77	32.14
Djibouti	25.57	51.33	25.77
Georgia	25.39	80.98	55.60
Togo	25.23	75.64	50.42
China	24.22	60.13	35.91
El Salvador	23.82	50.98	27.16
Ecuador	23.40	63.89	40.48
Liberia	22.35	69.61	47.26
Thailand	22.07	34.19	12.11
Qatar	21.48	30.03	8.55
Sao Tome and Principe	21.20	35.60	14.40
Iran	20.81	59.60	38.79

### 4.3. Analysis of Residuals

**Figure 3:**
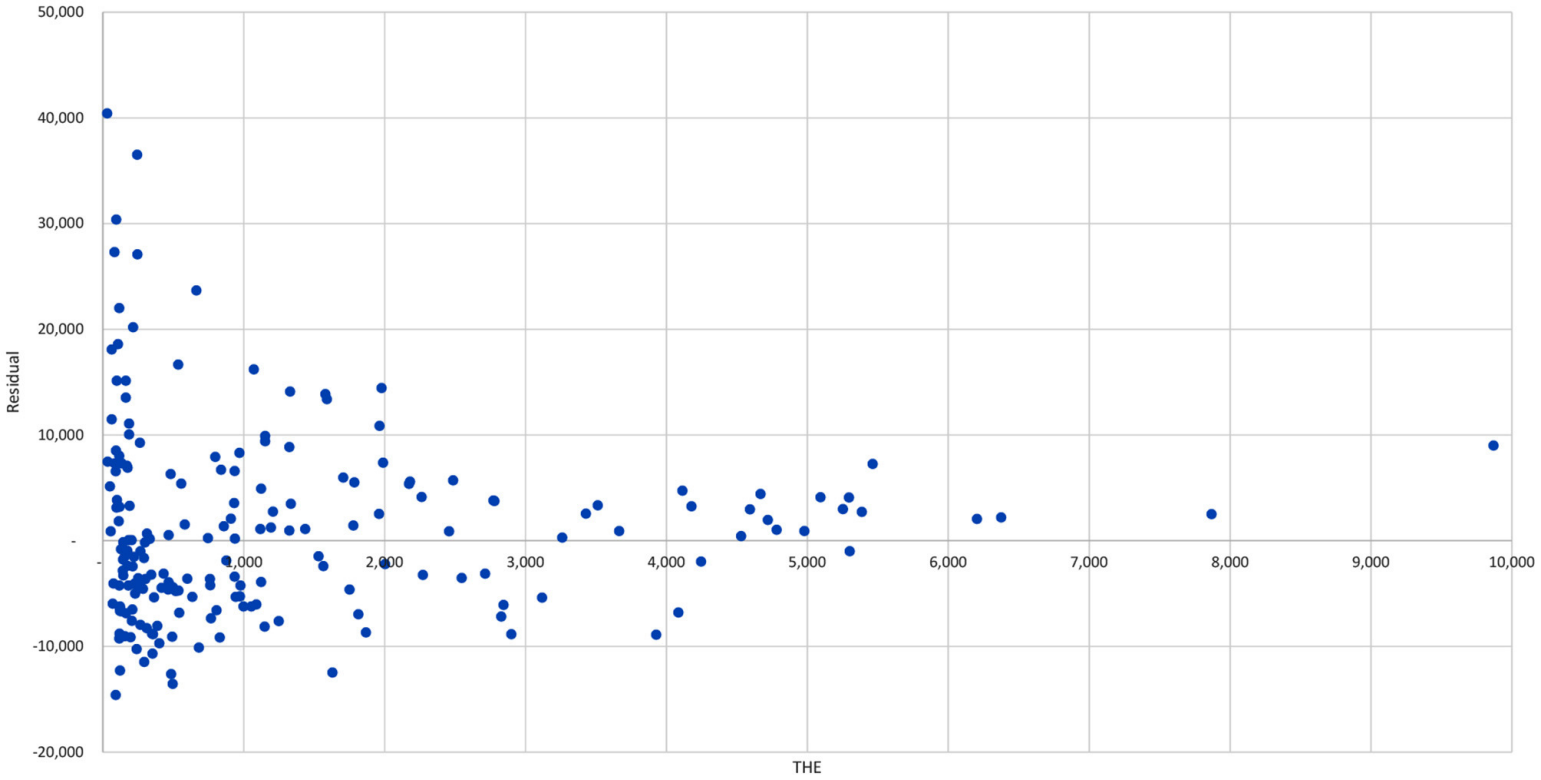
Residual vs. *THE* for 2016.

Based on the elasticities arrived at in Table 2 residuals ($\hat{\epsilon_{i}}$) are estimated for each country using Equation (7) and are listed in **Tables 11**–**15** for all the countries and regions in the data set. Figure 3 graphs the value of these residuals against *THE*. It can be seen from the graph that at each and every level of *THE* there are both positive and negative outliers. It is important to note though that at lower levels of *THE* the range of over/under-performance is far greater than at higher level of *THE*. This suggests that there may be useful additional lessons to gained from a careful analysis of these residuals, particularly for low-income countries, which would result in a large improvement in their health outcomes.

In order to aid in the analysis of these residuals, the health systems of countries are classified in this paper into different categories based on the criteria listed below (determined by the author based on where the *natural breakpoints* in health outcomes appeared to exist):

1. **Extent of Pooling**: *Pooled Dominant* if *OOP* < 25%; *Market Significant* if 25% < *OOP* < 50%; and *Market Dominant* if *OOP*> 50%.
2. **Health System Performance**: *High Performance* if *DALY* < 25, 000; *Medium Performance* if 25, 000 < *DALY* < 30, 000; and *Low Performance* if *DALY*> 30, 000.
3. **Extent of Outperformance**: *Positive Outlier* if Residual < −5, 000; *Negative Outlier* if Residual > 5000; and *Neutral* if −5000 < Residual < 5000.
4. **Size of Country**: *Small* if Population < 1 million; *Large* if Population > 1 million.

Using these and other categorizations, the interest is in studying countries and regions that operate in different environments, to understand what additional steps larger countries with high and medium performance health systems have taken to become *Positive Outliers* and what challenges are being experienced by the *Negative Outliers*. It is clear from the Tables 4–**10** that outlier countries can be drawn from any mix of pooled and out-of-pocket expenditures and *THE* levels.

**Table 4:** Pooled dominant (OOP < 25%) positive outlier countries.

**Country**	**THE**	**OOP%**	**DALY rate**	**Residual**	**Population**
Turkey	1,089	16.47	23,716	−6,024	79,821,724
Colombia	830	20.16	21,613	−9,153	48,171,392
Costa Rica	1,249	22.14	21,234	−7,584	4,899,345
Saudi Arabia	3,117	14.34	20,105	−5,373	32,442,572
Oman	2,827	5.91	19,821	−7,174	4,479,219
Israel	2,843	22.97	19,331	−6,059	8,546,000
Kuwait	2,899	16.11	16,795	−8,843	3,956,873
Qatar	3,926	8.55	16,313	−8,883	2,654,374
Thailand	635	12.11	27,412	−5,306	68,971,331

Table 4 lists large, high and medium performance countries which are *Pooled Dominant* (i.e., *OOP* < 25%). While each of these countries is worthy of careful study, of most immediate relevance in a developing country context would perhaps be Colombia (shaded in gray) which has been able to generate a *DALY Rate* of 21,613 against an expected rate, given its low *THE* level of $830, of 30,766, giving a high residual of -9,153. Amongst these high performing outliers, with a residual of −5,306 at a *THE* of only $635, while Thailand's achievements are indeed notable, its *DALY Rate*, at 27,412, still remains considerably above 20,000, suggesting that it still has long way to go before it can catch-up with the truly high performing countries list in the Table 4.

Table 5 lists large, high and medium performance countries which are *Market Significant* (i.e., 25% < *OOP* < 50%). Of particular interest in this list are Honduras, Peru, Nicaragua, and Jordan. Amongst these countries, Peru, Nicaragua, and Jordan (all shaded in gray), have *DALY Rates* close to or below 20,000 despite low *THE* levels and *OOP* > 25%. Honduras (shaded in gray) has a *THE* of only $400 and an *OOP* of 45% but has nevertheless been able to deliver a *DALY Rate* of <25,000.

**Table 5:** Market significant (25% < OOP < 50%) positive outlier countries.

**Country**	**THE**	**OOP%**	**DALY rate**	**Residual**	**Population**
Ecuador	943	40.48	24,474	-5,307	16,491,115
Mexico	972	40.38	24,390	-5,254	123,333,376
Honduras	400	45.01	24,290	−9,691	9,270,795
Tunisia	806	39.90	23,936	−6,562	11,303,946
Paraguay	768	37.86	23,388	−7,336	6,777,872
Algeria	998	30.88	23,360	−6,215	40,551,404
Malaysia	1,053	37.60	23,072	−6,209	30,684,804
Peru	681	28.29	21,305	−10,098	30,926,032
Lebanon	1,147	32.14	20,822	−8,114	6,711,121
Nicaragua	485	32.22	20,390	−12,610	6,303,974
Jordan	495	27.98	19,449	−13,529	9,551,467
Bahrain	1,866	27.99	18,277	−8,656	1,425,791
Singapore	4,084	31.17	17,066	−6,787	5,607,283
Indonesia	363	37.34	29,105	−5,342	261,554,226
Himachal Pradesh	266	49.50	28,320	−7,945	7,500,000
Vietnam	356	44.57	25,748	−8,836	93,638,724

Table 6 lists large, high and medium performance countries which are *Market Dominant* (i.e., *OOP*> 50%). All the countries / regions in this list are interesting, but in particular Sri Lanka and the Kyrgyz Republic (both shaded in gray). Sri Lanka is the only country with an *OOP* level that exceeds 50% that has been able to reduce its *DALY Rate* to <25,000. The Kyrgyz Republic spends only $240, with close to 60% of it being out-of-pocket, both numbers very similar to those of the Indian State of Tamil Nadu (**Table 10**), but, at 26,864, has been able to deliver a *DALY Rate* of close to 25,000, while, at 33,527, Tamil Nadu is well above 30,000.

**Table 6:** Market dominant (OOP > 50%) positive outlier countries.

**Country**	**THE**	**OOP%**	**DALY rate**	**Residual**	**Population**
Sri Lanka	491	50.12	23,965	−9,064	21,203,000
Bangladesh	91	71.89	29,601	−14,576	157,970,840
Kerala	386	71.30	27,301	−8,056	36,600,000
Kyrgyz Republic	240	57.59	26,864	−10,240	6,079,500

In Table 7 are countries that have high *THE* but all are doing more poorly that would have been expected, given their high *THE* > $1,000 and relatively low *OOP* levels. In this list while the presence of United States (shaded in gray), with its extraordinarily high *THE* = $9,870 is not entirely surprising, the fact that Germany (shaded in gray) should have a DALY Rate 30,000 when, given its *THE* = $5,463 and *OOP* = 12%, it was expected to have a *DALY Rate* of <25,000 with a very high residual of 7,283, does invite special attention. France, a very similar country, by contrast (**Table 9**) with a lower *THE* = $4,782 and *OOP* = 10% has a much lower *DALY Rate* of 25,328.

**Table 7:** Negative outlier countries with *THE* > $1,000.

**Country**	**THE**	**OOP%**	**DALY rate**	**Residual**	**Population**
United States	9,870	11	30,626	9,007	323,071,342
Germany	5,463	12	30,820	7,283	82,348,669
Czech Republic	2,485	15	32,044	5,720	10,566,332
Trinidad and Tobago	2,181	40	31,806	5,600	1,377,564
Slovak Republic	2,172	18	32,064	5,378	5,430,798
Estonia	1,988	23	34,220	7,395	1,315,790
Lithuania	1,978	32	41,068	14,442	2,868,231
Hungary	1,963	30	37,560	10,866	9,814,023
Poland	1,784	23	32,781	5,521	37,970,087
Croatia	1,705	15	33,836	5,982	4,174,349
Latvia	1,590	45	40,940	13,410	1,959,537
Bulgaria	1,578	48	41,485	13,878	7,127,822
Russian Federation	1,329	40	42,375	14,114	144,342,396
Serbia	1,323	41	37,145	8,862	7,058,322
Romania	1,152	21	38,643	9,408	19,702,332
Belarus	1,151	36	38,811	9,922	9,501,534
South Africa	1,071	8	47,085	16,219	56,203,654

Table 8 lists countries with *THE* < $100. All of them, with the exception of Bangladesh (shaded in gray) and Ethiopia, have a *DALY Rate* that exceeds 40,000. This is not in and of itself surprising but what is noteworthy is that, with the exception of Bangladesh, Ethiopia, and Gambia, they are all under-performing even relative to the low levels expected of them given their low *THE* and high *OOP%* levels.

**Table 8:** Very poor countries with *THE* < $100.

**Country**	**THE**	**OOP%**	**DALY rate**	**Residual**	**Population**
Central African Republic	30	43	90,879	40,441	4,537,687
Congo, Dem. Rep	34	37	56,802	7,484	78,789,127
Burundi	50	31	51,811	5,148	10,487,998
Eritrea	55	59	47,409	907	3,213,972
Niger	61	59	63,847	18,113	20,788,838
Mozambique	62	8	59,234	11,500	27,829,942
Ethiopia	70	37	38,364	−5,954	103,603,501
Gambia	74	24	40,188	−4,041	2,149,139
Mali	81	35	70,608	27,314	17,965,429
Benin	83	43	50,448	7,314	10,872,067
Madagascar	90	22	49,582	6,589	24,894,380
Bangladesh	91	72	29,601	−14,576	157,970,840
Papua New Guinea	92	8	53,372	8,538	8,271,760
Chad	95	61	73,341	30,391	14,561,666
Haiti	95	42	45,388	3,146	10,839,970
Guinea-Bissau	98	35	57,206	15,137	1,782,437
Togo	100	50	45,978	3,855	7,509,952

In Table 9, all of the countries with *THE* > $2,500 are listed. As a group it is clear from the table that they are perhaps either not getting the value from all their expenditures or there are factors within their economies, such as a rapidly aging population, that needs them to spend much more than they are currently doing to get better *DALY Rates*. However, in this group, France, Australia, Singapore, Spain, Saudi Arabia, Israel, South Korea, and United Arab Emirates (all shaded in gray) stand-out because either they are staying close to what is expected of them or doing much better, despite having problems comparable to those of other developed countries. France and Spain stand out as the only large European countries in the list, and South Korea as being perhaps one which is getting the most value for the relatively low *THE* = $2, 712 that it is spending. The countries of the middle-east, as a group, appear to be outperforming the other developed nations.

**Table 9:** Rich countries with *THE* > $2, 500.

**Country**	**THE**	**OOP%**	**DALY rate**	**Residual**	**Population**
United States	9,870	11	30,626	9,007	323,071,342
Switzerland	7,867	30	24,137	2,535	8,373,338
Norway	6,203	15	25,008	2,080	5,234,519
Germany	5,463	12	30,820	7,283	82,348,669
Sweden	5,387	15	26,106	2,727	9,923,085
Ireland	5,300	13	22,625	−974	4,755,335
Austria	5,295	19	27,345	4,102	8,736,668
Netherlands	5,251	11	26,766	2,998	17,030,314
Denmark	5,093	14	27,811	4,125	5,728,010
France	4,782	10	25,328	1,036	66,859,768
Canada	4,718	15	25,872	1,974	36,109,487
Belgium	4,668	16	28,299	4,442	11,331,422
Japan	4,592	13	27,062	2,979	126,994,511
Australia	4,530	19	24,239	437	24,190,907
United Kingdom	4,178	15	27,570	3,259	65,595,565
Finland	4,112	20	28,834	4,740	5,495,303
Singapore	4,084	31	17,066	−6,787	5,607,283
Qatar	3,926	9	16,313	−8,883	2,654,374
New Zealand	3,665	14	25,831	915	4,693,200
Italy	3,427	23	27,239	2,567	60,627,498
Spain	3,260	24	25,153	314	46,483,569
Saudi Arabia	3,117	14	20,105	−5,373	32,442,572
Kuwait	2,899	16	16,795	−8,843	3,956,873
Israel	2,843	23	19,331	−6,059	8,546,000
Oman	2,827	6	19,821	−7,174	4,479,219
Portugal	2,778	28	29,116	3,764	10,325,452
Slovenia	2,772	12	29,950	3,807	2,065,042
South Korea	2,712	33	22,270	−3,096	51,245,707
United Arab Emirates	2,546	19	22,484	−3,524	9,360,980

It is interesting to note from Table 10 that the Indian states as a group are broadly doing as well as can be expected, but given their very low *THE* levels (*Neutral Performance* with −5, 000 < *Residual* < 5, 000) and very high *OOP%* levels they have *DALY Rates* well above 30,000. The only two exceptions being the states of Kerala and Himachal Pradesh (both shaded in gray along with India). Kerala in particular is note worthy because while it has a low *THE* = $386, and a verylarge *OOP%* = 71%, it nevertheless appears to have been able to harness market forces to deliver a globally respectable *DALY Rate* of 27,301.

**Table 10:** Indian states.

**State**	**THE**	**OOP%**	**DALY rate**	**Residual**	**Population**
Bihar	120	80	37,074	−6,347	108,100,000
Jharkhand	122	66	35,095	−6,630	35,700,000
Assam	129	55	39,915	−784	33,900,000
Madhya Pradesh	145	70	37,678	−3,284	77,900,000
Uttar Pradesh	174	77	39,585	−943	218,400,000
Rajasthan	174	56	36,556	−2,368	74,790,000
Gujarat	180	50	34,291	−4,227	66,100,000
Chhattisgarh	182	58	38,810	61	28,200,000
Odisha	203	72	39,091	70	44,900,000
Jammu and Kashmir	206	56	30,363	−7,561	13,900,000
Uttarakhand	211	61	35,622	−2,416	10,280,000
Haryana	220	60	36,191	−1,507	27,600,000
India	222	61	35,435		1,320,000,000
Andhra Pradesh	224	75	34,721	−4,066	52,500,000
Tamil Nadu	234	65	33,527	−4,154	77,800,000
Maharashtra	255	59	32,677	−4,147	119,600,000
Himachal Pradesh	266	50	28,320	−7,945	7,500,000
Karnataka	266	50	35,277	−979	66,000,000
Telangana	284	58	31,646	−4,532	38,600,000
Punjab	302	77	33,766	−3,606	29,600,000
Kerala	386	71	27,301	−8,056	36,600,000

## 5. Discussion

Policy makers in developing countries have to work within severe resource constraints and need to deploy them with care in order to achieve their multiple policy goals. Improving the performance of their health systems is an urgent imperative for them and any systematic insights that they can gather from the experiences of other countries, both high and low performing, are likely to be of great value. This study analyses the performance of health systems around the world to understand more precisely the respective roles of total health expenditures, pooling of these expenditures, and multiple other factors, in shaping the behavior of health systems.

It is already well-known from the literature that total health expenditures and the extent of pooling matters (3, 27) for developing country health systems. From this study we learn additionally that while total expenditure on health does indeed matter, beyond a minimum level, it is neither necessary nor sufficient and can perhaps be excessive as well. From Table 1, it can be seen that there is a robust estimate of elasticity (γ) of −0.1557 associated with *THE*. This can also be seen from Figure 2 which indicates that there is a clear negative association between *THE* and the *DALY Rate*. However, from both Table 1 and Figure 2, it can clearly be seen that there are many countries with both high and low *THE* levels that are doing much worse and much better than others in their cohort. Tables 4–10 also bear this out and suggest that, while there may be a minimum level of *THE* > $100 which may need to be crossed for a health system to perform (Bangladesh being a clear and sole exception), it is possible for both countries and states with low levels of *THE* to perform very well (such as Colombia, Thailand Honduras, Peru, Nicaragua, Jordan, Sri Lanka, and the Krygyz Republic), and for those with high levels of *THE* to under-perform (such as United States and Germany).

Tables 4–6 reaffirm the insight that the extent of pooling matters, but go on to make the point that very high rates (> 75%) of pooling are essential to building truly high performing health systems (with *DALYRates* < 20, 000). It is also apparent from the tables that merely having a high level of pooling on its own is insufficient to deliver strong health outcomes, and also that even at lower levels of pooling it is possible to out-perform one's peers using other Control Knobs (Figure 1). From Table 2 it can be seen that the elasticity associated with pooled expenditures (α = −0.0941) is almost double that of the one associated with out-of-pocket expenditures (β = −0.0584), with both having p-values that are well below the 1% level. So clearly the more the level of pooling, on average, the better the outcomes but from Figure 3 it can be seen that there are a number of countries that are doing much better than their level of pooling would imply. This can be seen more clearly from Tables 5–7 where, while many countries with high levels of pooling (and high levels of *THE*) are doing poorly, others such as the Ecuador, Mexico, Honduras, Malaysia, Vietnam, Kyrgyz Republic, and Sri Lanka are doing very well despite having out-of-pocket expenditures in the region of 40–60%. While a measure of pooling (in any form) is beneficial, the manner in which pooling and associated purchasing arrangements are setup does matter a great deal to get high performance. A review of the performance of the countries listed in Tables 4–9, such as Colombia, Costa Rica, Israel, Thailand, United States, Germany, France, Australia, and Spain suggests that while pooling (in any form) is definitely beneficial, countries with single payer systems are perhaps more effective than those with multiple payers perhaps because, despite their best efforts, the multi-payer countries have insufficient market power over customers and providers to adequately manage pulls and pressures of market forces. This hypothesis is also consistent with the arguments made in (28) regarding inflation rates associated with different health system arrangements.

From the list of *Positive Outliers*, it can also be gathered that, consistent with existing insights (29), an emphasis on strong provision of essential public health services by the government can result in low *DALY Rates* even at low *THE*. Countries and regions such as Honduras, Peru, Nicaragua, Jordan, Sri Lanka, Bangladesh, Kerala, and the Kyrgyz Republic (Tables 5, 6) despite modest levels of *THE* have delivered attractive *DALY Rates* on account of their consistent prioritization of public-health interventions such as near 100% vaccine coverage levels and strong control of infectious diseases. From the examples of Turkey, Colombia, Costa Rica, Thailand, Peru, Nicaragua, and Jordan in Tables 4, 5, which have all delivered low *DALY Rates* despite modest levels of *THE*, it can also be seen that an emphasis on primary care, another well known insight (30, 31), can result in low *DALY Rates* even at low *THE*.

While, as can be seen from the discussion, several valuable conclusions can be drawn from this kind of analysis, the evolution of health systems is a complex journey, driven by multiple local factors, and a multi-country cross-sectional study of the type attempted here runs the risk of glossing over them (18). There are also multiple concerns about the methodologies associated with the computation of *DALYs* (19–21). The study attempts to address these limitations by being parsimonious and simple in its approach toward specifying its quantitative models and validating its conclusions by looking deeper into country contexts. However, another, related, limitation of the study is that while it has indeed made an attempt to examine the experiences of outlier countries, it has not done so at the level of depth that would be needed—additional research to address this shortcoming could yield powerful insights. Policy makers and researchers interested in these insights would do well to keep these limitations of the study in mind while reviewing the conclusions presented here (Tables 11–15).

**Table 11:** Estimated residuals for each country (Afghanistan to Republic of the Congo).

**Country**	**THE (PPP$)**	**OOP%**	**DALY rate**	**Residual**	**Population**
Afghanistan	163	77	56,197	15,136	35,383,128
Albania	760	58	27,533	−3,611	2,876,101
Algeria	998	31	23,360	−6,215	40,551,404
Andorra	4,979	42	24,032	921	77,297
Angola	186	35	48,217	10,059	28,842,484
Antigua and Barbuda	976	32	25,420	−4,236	94,527
Argentina	1,531	16	26,815	−1,467	43,590,368
Armenia	877	81	30,296	−1,855	2,936,146
Australia	4,530	19	24,239	437	24,190,907
Austria	5,295	19	27,345	4,102	8,736,668
Azerbaijan	1,193	79	31,728	1,259	9,757,812
Bahamas, The	1,436	28	29,157	1,118	377,931
Bahrain	1,866	28	18,277	−8,656	1,425,791
Bangladesh	91	72	29,601	−14,576	157,970,840
Barbados	1,323	45	29,296	976	285,796
Belarus	1,151	36	38,811	9,922	9,501,534
Belgium	4,668	16	28,299	4,442	11,331,422
Belize	541	23	25,903	−6,799	368,400
Benin	83	43	50,448	7,314	10,872,067
Bhutan	293	20	24,603	−11,456	736,709
Bolivia	496	28	28,572	−4,390	11,031,813
Bosnia and Herzegovina	1,123	29	34,011	4,925	3,386,267
Botswana	931	5	35,756	3,578	2,159,944
Brazil	1,777	44	28,514	1,458	206,163,058
Brunei Darussalam	1,812	5	22,165	−6,959	419,800
Bulgaria	1,578	48	41,485	13,878	7,127,822
Burkina Faso	116	31	63,087	22,011	18,646,378
Burundi	50	31	51,811	5,148	10,487,998
Cabo Verde	348	26	26,080	−8,781	531,146
Cambodia	229	59	32,434	−4,998	15,766,293
Cameroon	169	70	47,025	7,093	23,926,539
Canada	4,718	15	25,872	1,974	36,109,487
Central African Republic	30	43	90,879	40,441	4,537,687
Chad	95	61	73,341	30,391	14,561,666
Chile	2,002	35	24,356	−2,201	18,209,068
China	761	36	26,553	−4,216	13,786,65,000
Colombia	830	20	21,613	−9,153	48,171,392
Comoros	116	73	33,458	−9,232	795,592
Congo, Dem. Rep.	34	37	56,802	7,484	78,789,127
Congo, Rep.	263	50	45,587	9,269	4,980,999

**Table 12:** Estimated residuals for each country (Costa Rica to Israel).

**Country**	**THE (PPP$)**	**OOP%**	**DALY rate**	**Residual**	**Population**
Costa Rica	1,249	22	21,234	−7,584	4,899,345
Cote d'Ivoire	163	40	52,484	13,551	23,822,714
Croatia	1,705	15	33,836	5,982	4,174,349
Cuba	2,458	10	27,728	911	11,335,109
Cyprus	2,271	45	22,844	−3,233	1,170,187
Czech Republic	2,485	15	32,044	5,720	10,566,332
Denmark	5,093	14	27,811	4,125	5,728,010
Djibouti	122	26	34,690	−6,214	929,112
Dominica	581	29	33,700	1,544	71,307
Dominican Republic	937	45	30,063	220	10,397,743
Ecuador	943	40	24,474	−5,307	16,491,115
Egypt, Arab Rep.	516	62	28,456	−4,760	94,447,072
El Salvador	600	27	28,455	−3,592	6,356,143
Equatorial Guinea	839	73	38,255	6,715	1,215,179
Eritrea	55	59	47,409	907	3,213,972
Estonia	1,988	23	34,220	7,395	1,315,790
Eswatini	663	10	56,493	23,683	1,113,984
Ethiopia	70	37	38,364	−5,954	103,603,501
Fiji	313	21	36,347	680	872,399
Finland	4,112	20	28,834	4,740	5,495,303
France	4,782	10	25,328	1,036	66,859,768
Gabon	556	23	37,991	5,402	2,007,873
Gambia, The	74	24	40,188	−4,041	2,149,139
Georgia	797	56	38,764	7,932	3,727,505
Germany	5,463	12	30,820	7,283	82,348,669
Ghana	189	38	41,338	3,302	28,481,946
Greece	2,261	34	30,217	4,146	10,775,971
Grenada	745	58	31,481	252	110,261
Guatemala	462	53	28,825	−4,602	16,583,060
Guinea	108	50	60,225	18,603	11,738,441
Guinea-Bissau	98	35	57,206	15,137	1,782,437
Guyana	333	35	35,091	175	771,366
Haiti	95	42	45,388	3,146	10,839,970
Honduras	400	45	24,290	−9,691	9,270,795
Hungary	1,963	30	37,560	10,866	9,814,023
Iceland	4,245	17	22,179	−1,966	335,439
Indonesia	363	37	29,105	−5,342	261,554,226
Iran, Islamic Rep.	1,564	39	25,164	−2,402	79,564,016
Ireland	5,300	13	22,625	−974	4,755,335
Israel	2,843	23	19,331	−6,059	8,546,000

**Table 13:** Estimated residuals for each country (Italy to Niger).

**Country**	**THE (PPP$)**	**OOP%**	**DALY rate**	**Residual**	**Population**
Italy	3,427	23	27,239	2,567	60,627,498
Jamaica	536	22	28,079	−4,697	2,906,238
Japan	4,592	13	27,062	2,979	126,994,511
Jordan	495	28	19,449	−13,529	9,551,467
Kazakhstan	859	36	31,589	1,378	17,794,055
Kenya	144	28	38,057	−1,779	49,051,686
Kiribati	250	0.1	45,981	−3,545	112,524
Korea, Rep.	2,712	33	22,270	−3,096	51,245,707
Kuwait	2,899	16	16,795	−8,843	3,956,873
Kyrgyz Republic	240	58	26,864	−10,240	6,079,500
Lao PDR	155	46	37,840	−1,471	6,845,846
Latvia	1,590	45	40,940	13,410	1,959,537
Lebanon	1,147	32	20,822	−8,114	6,711,121
Lesotho	243	19	73,714	36,518	2,075,001
Liberia	133	47	47,553	7,317	4,586,788
Lithuania	1,978	32	41,068	14,442	2,868,231
Luxembourg	6,374	11	25,303	2,207	582,014
Madagascar	90	22	49,582	6,589	24,894,380
Malawi	115	11	50,614	8,045	17,205,289
Malaysia	1,053	38	23,072	−6,209	30,684,804
Maldives	1,629	19	15,345	−12,468	475,513
Mali	81	35	70,608	27,314	17,965,429
Malta	3,511	35	27,732	3,355	455,356
Marshall Islands	934	9	37,881	6,598	57,735
Mauritania	164	51	32,227	−6,847	4,163,534
Mauritius	1,207	48	31,518	2,754	1,263,473
Mexico	972	40	24,390	−5,254	123,333,376
Micronesia, Fed. Sts.	432	3	34,477	−3,099	110,215
Moldova	480	46	39,388	6,321	3,551,954
Mongolia	467	36	33,704	550	3,056,359
Montenegro	1,334	24	31,948	3,491	622,303
Morocco	466	49	29,350	−3,918	35,126,296
Mozambique	62	8	59,234	11,500	27,829,942
Myanmar	291	74	35,549	−1,632	53,045,226
Namibia	969	8	39,678	8,330	2,358,041
Nepal	156	55	30,504	−9,030	27,261,131
Netherlands	5,251	11	26,766	2,998	17,030,314
New Zealand	3,665	14	25,831	915	4,693,200
Nicaragua	485	32	20,390	−12,610	6,303,974
Niger	61	59	63,847	18,113	20,788,838

**Table 14:** Estimated residuals for each country (Nigeria to Tonga).

**Country**	**THE (PPP$)**	**OOP%**	**DALY rate**	**Residual**	**Population**
Nigeria	214	75	59,325	20,210	185,960,289
North Macedonia	935	35	26,423	−3,402	2,080,745
Norway	6,203	15	25,008	2,080	5,234,519
Oman	2,827	6	19,821	−7,174	4,479,219
Pakistan	144	65	40,444	−128	203,627,284
Panama	1,750	27	22,593	−4,617	4,037,078
Papua New Guinea	92	8	53,372	8,538	8,271,760
Paraguay	768	38	23,389	−7,336	6,777,872
Peru	681	28	21,305	−10,098	30,926,032
Philippines	342	54	31,805	−3,211	103,663,927
Poland	1,784	23	32,781	5,521	37,970,087
Portugal	2,778	28	29,116	3,764	10,325,452
Qatar	3,926	9	16,313	−8,883	2,654,374
Romania	1,152	21	38,643	9,408	19,702,332
Russian Federation	1,329	40	42,375	14,114	144,342,396
Rwanda	130	6	36,319	−6,667	11,668,818
Samoa	353	12	25,156	−10,662	194,535
Sao Tome and Principe	197	14	29,688	−9,130	203,227
Saudi Arabia	3,117	14	20,105	−5,373	32,442,572
Senegal	142	52	37,151	−2,828	14,993,528
Serbia	1,323	41	37,145	8,862	7,058,322
Seychelles	1,123	2	29,014	−3,891	94,677
Sierra Leone	244	42	63,705	27,100	7,328,838
Singapore	4,084	31	17,066	−6,787	5,607,283
Slovak Republic	2,172	18	32,064	5,378	5,430,798
Slovenia	2,772	12	29,950	3,807	2,065,042
Solomon Islands	118	5	35,631	−8,788	619,437
South Africa	1,071	8	47,085	16,219	56,203,654
Spain	3,260	24	25,153	314	46,483,569
Sri Lanka	491	50	23,965	−9,064	21,203,000
Sudan	298	74	36,887	−155	39,847,440
Suriname	908	22	32,370	2,102	564,888
Sweden	5,387	15	26,106	2,727	9,923,085
Switzerland	7,867	30	24,137	2,535	8,373,338
Tajikistan	209	66	31,904	−6,505	8,663,579
Tanzania	112	22	43,488	1,845	53,050,790
Thailand	635	12	27,412	−5,306	68,971,331
Timor−Leste	122	9	30,445	−12,274	1,219,288
Togo	100	50	45,978	3,855	7,509,952
Tonga	311	11	28,390	−8,249	101,133

**Table 15:** Estimated residuals for each country (Trinidad and Tobago to Zimbabwe; India and Indian States).

**Country**	**THE (PPP$)**	**OOP%**	**DALY rate**	**Residual**	**Population**
Trinidad and Tobago	2,181	40	31,806	5,600	1,377,564
Tunisia	806	40	23,936	−6,562	11,303,946
Turkey	1,089	16	23,716	−6,024	79,821,724
Turkmenistan	1,117	76	31,611	1,121	5,662,372
Uganda	117	40	44,149	3,215	39,647,506
Ukraine	534	54	49,397	16,667	45,004,645
United Arab Emirates	2,546	19	22,484	−3,524	9,360,980
United Kingdom	4,178	15	27,570	3,259	65,595,565
United States	9,870	11	30,626	9,007	323,071,342
Uruguay	1,959	17	29,676	2,540	3,424,132
Uzbekistan	417	52	29,492	−4,434	31,847,900
Vanuatu	116	8	38,921	−4,218	278,330
Vietnam	356	45	25,748	−8,836	93,638,724
Zambia	175	12	46,731	6,914	16,363,507
Zimbabwe	185	21	49,702	11,093	14,030,390
**India**	222	61	35,435		1,320,000,000
Andhra Pradesh	224	75	34,721	−4,066	52,500,000
Assam	129	55	39,915	−784	33,900,000
Bihar	120	80	37,074	−6,347	108,100,000
Chhattisgarh	182	58	38,810	61	28,200,000
Gujarat	180	50	34,291	−4,227	66,100,000
Haryana	220	60	36,191	−1,507	27,600,000
Himachal Pradesh	266	50	28,320	−7,945	7,500,000
Jammu and Kashmir	206	56	30,363	−7,561	13,900,000
Jharkhand	122	66	35,095	−6,630	35,700,000
Karnataka	266	50	35,277	−979	66,000,000
Kerala	386	71	27,301	−8,056	36,600,000
Madhya Pradesh	145	70	37,678	−3,284	77,900,000
Maharashtra	255	59	32,677	−4,147	119,600,000
Odisha	203	72	39,091	70	44,900,000
Punjab	302	77	33,766	−3,606	29,600,000
Rajasthan	174	56	36,556	−2,368	74,790,000
Tamil Nadu	234	65	33,527	−4,154	77,800,000
Telangana	284	58	31,646	-4,532	38,600,000
Uttar Pradesh	174	77	39,585	-943	218,400,000
Uttarakhand	211	61	35,622	-2,416	10,280,000

## 6. Conclusion

There is a generally accepted view that higher levels of total health expenditure (*THE*) in a country lead to better health outcomes, particularly if spent using pooled instead of out-of-pocket expenditures. In this paper, using *DALY Rates* as an outcome indicator, the effects of *THE*, and pooled (*POOL*) and out-of-pocket expenditures (*OOP*) are examined using simple Cobb-Douglas health-production functions. Consistent with the accepted view, this analysis indicates that for every 1% increase in *THE, DALY Rates* fall by 0.15% and that a 1% increase in pooled expenditures reduces *DALY Rates* by 0.095% while a similar increase in out-of-pocket expenditure, at 0.06%, leads to a much lower quantum of reduction in *DALY Rates*.

However, the analysis also indicates that these variables are able to explain less than 50% of the variation in *DALY Rates*, leaving fairly large *unexplained* residuals. An analysis of these residuals suggests several interesting insights, which bear further scrutiny. The analysis, for example, clearly indicates that developing countries which are able to spend in excess of ≈$100 per-capita can aspire to good health outcomes for their citizens and do not necessarily need to wait for several decades for national per-capita income to grow to a level that allows them to considerably increase their aggregate spending on health as a country. However, there are several other steps that they would need to take to *produce* good health from their current levels of health expenditures. These include making an effort to increase the level of pooling to > 75%, and moving in a direction such that a single payer is responsible for purchasing healthcare with these pooled resources. Additionally, all countries, including those with low levels of total health expenditure, need to ensure that their governments first properly complete the task of providing public-health public/merit-goods such as vaccinations and infectious disease control if they wish to have good health outcomes. And, whether using pooled funds or out-of-pocket expenditures, they need to be aware that a strong emphasis on comprehensive primary care can result in low *DALY Rates* even at low levels of total health expenditures.

Good health outcomes are obtainable both with private provision and through the use of government-owned providers. However, even with high levels of total health expenditures combined with high levels of pooling, unless carefully designed purchasing arrangements are put in place, it is possible to deliver relatively poor health outcomes, and, conversely even a small amount of pooled resources, if spent in a catalytic manner, can help deliver strong outcomes in the overall health system by ensuring that citizens get better value for their out-of-pocket expenditures.

## Data Availability Statement

The original contributions presented in the study are included in the article/supplementary material, further inquiries can be directed to the corresponding author/s.

## Author Contributions

The author confirms being the sole contributor of this work and has approved it for publication.

## Conflict of Interest

The author declares that the research was conducted in the absence of any commercial or financial relationships that could be construed as a potential conflict of interest.

## Publisher's Note

All claims expressed in this article are solely those of the authors and do not necessarily represent those of their affiliated organizations, or those of the publisher, the editors and the reviewers. Any product that may be evaluated in this article, or claim that may be made by its manufacturer, is not guaranteed or endorsed by the publisher.

## Footnotes

^1^See (6) for a more detailed discussion on the computation of DALYs.

^2^These forces are similar to those found in physical systems and are referred to as the ones increasing the total entropy (23) of the system. This is an apt analogy for health systems where the *laissez-faire* approach toward the functioning of the system found in many developing country situations, with the government almost exclusively focused only on the providers and facilities it manages and owns, has led market forces to having free rein and producing highly distorted outcomes (24).
